# Partner, Neighbor, Housekeeper and Dimension: 3D versus 2D Glomerular Co-Cultures Reveal Drawbacks of Currently Used Cell Culture Models

**DOI:** 10.3390/ijms241210384

**Published:** 2023-06-20

**Authors:** Anna Rederer, Victoria Rose, René Krüger, Linda Schmittutz, Izabela Swierzy, Lena Fischer, Ingo Thievessen, Julian Bauer, Oliver Friedrich, Mario Schiffer, Janina Müller-Deile

**Affiliations:** 1 Department of Nephrology, Universitätsklinikum Erlangen, Friedrich-Alexander-Universität Erlangen-Nürnberg, 91054 Erlangen, Germany; anna.laptii@uk-erlangen.de (A.R.); victoria.rose@uk-erlangen.de (V.R.); rene.krueger@uk-erlangen.de (R.K.); linda.schmittzutz@gmail.com (L.S.); izabela.swierzy@uk-erlangen.de (I.S.); mario.schiffer@uk-eralngen.de (M.S.); 2 Center for Medicine, Physics and Technology, Friedrich-Alexander-Universität Erlangen-Nürnberg, 91054 Erlangen, Germany; lena.lf.fischer@fau.de (L.F.); ingo.thievessen@fau.de (I.T.); 3 Institute of Medical Biotechnology, Department of Chemical and Biological Engineering, Friedrich-Alexander-Universität Erlangen-Nürnberg, 91054 Erlangen, Germany; julian.bauer@fau.de (J.B.); oliver.friedrich@fau.de (O.F.)

**Keywords:** glomerular spheroids, bulk-RNA sequencing, 3D co-culture, cell culture model, cell matrix, cell–cell communication

## Abstract

Signaling-pathway analyses and the investigation of gene responses to different stimuli are usually performed in 2D monocultures. However, within the glomerulus, cells grow in 3D and are involved in direct and paracrine interactions with different glomerular cell types. Thus, the results from 2D monoculture experiments must be taken with caution. We cultured glomerular endothelial cells, podocytes and mesangial cells in 2D/3D monocultures and 2D/3D co-cultures and analyzed cell survival, self-assembly, gene expression, cell–cell interaction, and gene pathways using live/dead assay, time-lapse analysis, bulk-RNA sequencing, qPCR, and immunofluorescence staining. Without any need for scaffolds, 3D glomerular co-cultures self-organized into spheroids. Podocyte- and glomerular endothelial cell-specific markers and the extracellular matrix were increased in 3D co-cultures compared to 2D co-cultures. Housekeeping genes must be chosen wisely, as many genes used for the normalization of gene expression were themselves affected in 3D culture conditions. The transport of podocyte-derived VEGFA to glomerular endothelial cells confirmed intercellular crosstalk in the 3D co-culture models. The enhanced expression of genes important for glomerular function in 3D, compared to 2D, questions the reliability of currently used 2D monocultures. Hence, glomerular 3D co-cultures might be more suitable in the study of intercellular communication, disease modelling and drug screening ex vivo.

## 1. Introduction

Glomeruli form the functional units of the kidney responsible for selective blood filtration. The glomerular filtration barrier is composed of a tri-layered structure of fenestrated glomerular endothelial cells, the glomerular basement membrane, and podocytes building the slit diaphragm [1]. Another important component of glomeruli are mesangial cells whose matrix contributes to glomerular integrity and stability [2,3]. Intraglomerular cell communication between different cell types is essential for the integrity of the glomerular filtration barrier and also for glomerular function and structure [4,5,6,7]. In the past, most basic research questions on glomerular cells concerning signaling pathways, response to different stimuli, and gene expression were either studied in mouse in vivo or in 2D monocultures of conditionally immortalized cell lines in vitro [8]. Despite their known advantages, transgenic mouse models have high maintenance costs, are time-consuming and ethically questionable. In contrast, conventional 2D in vitro cell cultures are well-established, cost-effective, and easy to handle. However, they do not reflect the complexity of in vivo structures since the 3D architecture, intra- and cell–cell interaction and communication of the different glomerular cell types are missing. This results in an incomplete differentiation and a limited expression of cell type-specific markers, in particular podocyte markers [9]. Within the glomerulus, cells grow in a 3D environment with paracrine signaling of the glomerular cell types, which is not guaranteed in 2D monocultures. Data generated from classical 2D monocultures do not consider these limitations and, therefore, should be taken with caution.

Recently, some advanced approaches have been developed to better mimic the in vivo microenvironment [10]. They describe co- and 3D-cultivation as well as the generation of dynamic conditions. Innovative kidney models include on-a-chip platforms [11,12,13,14], scaffolds [15,16,17], hiPSC-derived cell models [14,18], organoids [19,20], biofabrication [21,22] and bioprinting [23]. The selection of the appropriate method depends on the specific research questions. To investigate intercellular interaction and create a more physiological but still simple microenvironment, 3D spheroidal cell aggregates or, simply, spheroids consisting of heterogenous cell populations have been exploited in the field of oncology, stem cell biology, and tissue engineering [24,25,26]. In these non-glomerular models, genes upregulated in 3D were associated with hypoxia, angiogenesis, inflammation, stress response, and redox signaling [27,28,29].

In basic nephrology research, it was reported that co-cultured glomerular spheroids of two [30] or three [31] cell types demonstrated enhanced cell maturation and extracellular matrix formation. However, these models either lacked mesangial cells, did not use human cell lines, inserted stem cells or applied artificial tools, such as magnetic beats, for 3D formation [21,31].

We inserted the three main glomerular cells types, namely human podocytes, human glomerular endothelial cells and mesangial cells, in glomerular 3D spheroids that formed by self-aggregation. This enabled the simulation of paracrine communication and cell–cell interaction between the different cell types. Furthermore, we compared glomerular co-cultures to monocultures in 2D and 3D culture by bulk-RNA sequencing and could identify differences in gene expression induced by culture conditions. By qPCR and immunofluorescence staining, increased expression of cell type-specific markers, extracellular matrix proteins and genes involved in cell–cell communication due to the co-culture situation or the cultivation in 3D could be validated. Finally, yet importantly, this analysis demonstrates that the normalization to typically used housekeeper genes, such as glyceraldehyde 3-phosphate dehydrogenase (*GAPDH*), hypoxanthine-guanine-phosphoribosyltransferase (*HPRT*),actin beta (*ACTB*), ubiquitin C (*UBC*) and succinate dehydrogenase complex flavoprotein subunit A (*SDHA*), is not applicable when comparing 2D and 3D culture models, as these genes are themselves regulated by th ese culture conditions.

## 2. Results

### 2.1. 3D Co-Culture of Human Glomerular Cell Types Improves Cell Survival

**Figure 1:**
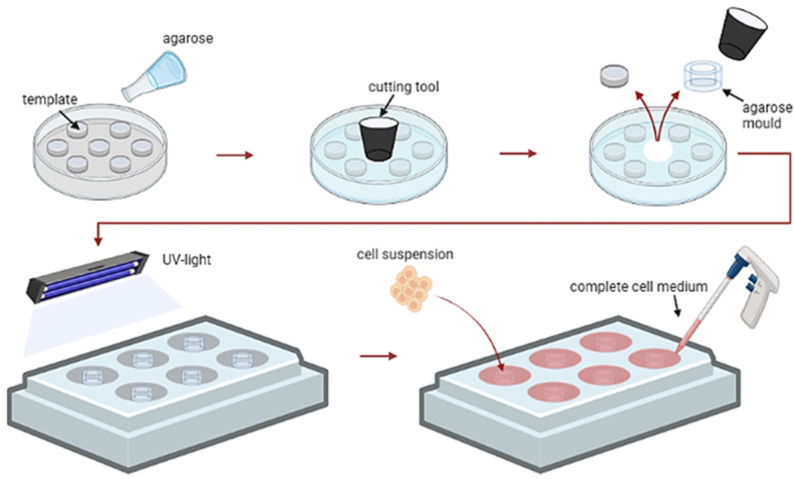
Schematic illustration of the generation of 3D spheroids by self-assembly of the cells in agarose micro-wells.

We cultured immortalized human podocytes, human primary glomerular endothelial cells, human primary mesangial cells in individual monocultures and in co-cultures of all three cell types in classical 2D culture conditions or in a 3D manner using custom-made agarose micro-wells (Figure 1).

**Figure 2:**
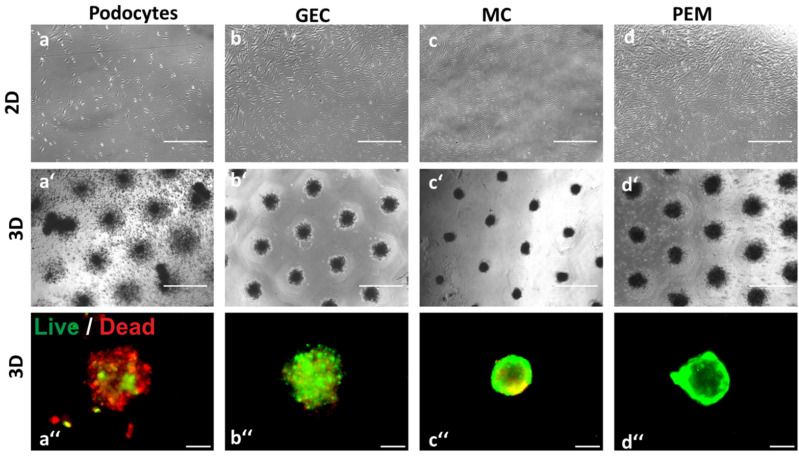
Mono- and co-cultures of differentiated, conditionally immortalized human podocytes (**a**); primary human glomerular endothelial cells (GEC) (**b**); primary human mesangial cells (MC) (**c**); and co-culture of all three glomerular cell types (PEM) (**d**) in 2D (**a**–**d**) or 3D (**a′**–**d′**) in agarose micro-wells display different morphology. Survival assay of spheroids (**a″**–**d″**). Living cells appear in green fluorescence and dead cells in red. Scale bar of (**a**–**d**,**a′**–**d′**) represents 750 µm and 100 µm for (**a″**–**d″**).

The 3D mono-and co-cultures self-aggregated into round spheroids within four days by gravity. However, the podocyte monocultures only formed instable spheroids. Cell survival of the different 3D cultures was assessed using a live/dead assay which revealed a significant number of dead cells in the 3D podocyte monoculture indicating that podocytes require other glomerular cell types to survive in 3D (Figure 2). Mesangial cell 3D monocultures and glomerular 3D cocultures got more compact over time whereas glomerular endothelial cell spheroids stayed the same over time (Supplementary Figure S1).

### 2.2. 3D Glomerular Co-Cultures Self-Arrange in a Specific Manner and Develop Fenestrae-like Structures of GEC and Interdigitating Cell Processes of Podocytes

**Figure 3:**
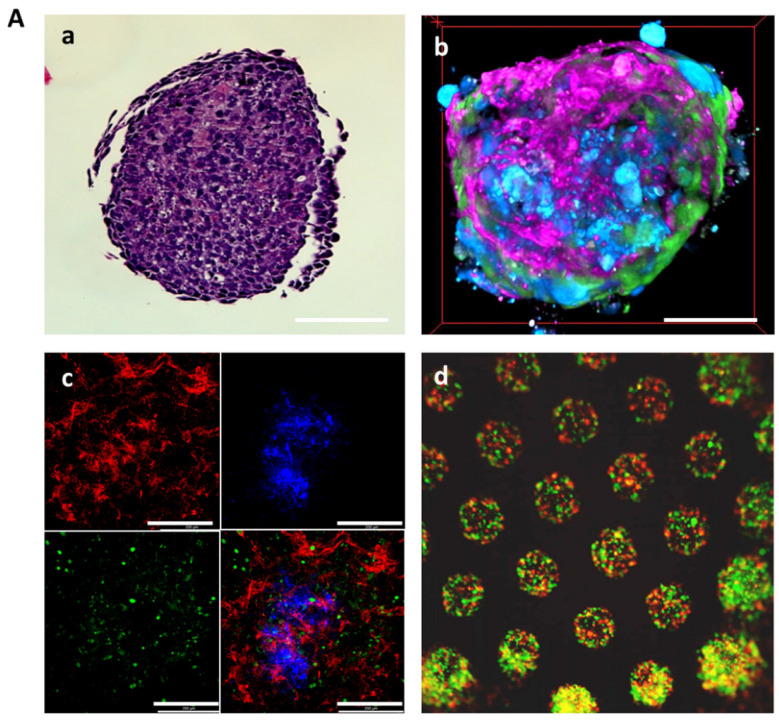

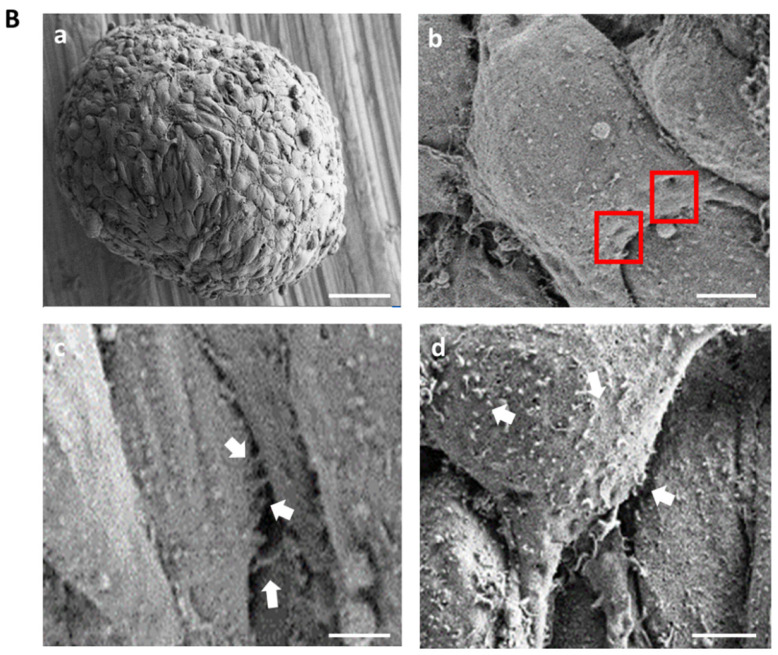
Self-organization of the 3D glomerular co-culture. (**A**) Self-assembly of 3D glomerular co-cultures: (**a**) HE stained section of the 3D glomerular co-culture. Scale bar represents 50 µm; (**b**) top-down view onto a three-dimensionally reconstructed multiphoton microscopy image stack of the glomerular 3D co-culture. Podocytes are labeled in blue, glomerular endothelial cells in magenta and mesangial cells in green. Scale bar represents 50 µm. An animated video is provided in Supplementary Video S2; and (**c**) confocal image of 3D glomerular co-culture. Podocytes (blue) cluster in the center and glomerular tdTomato-Farnesyl endothelial reporter cells (red) developed a reticular structure with mesangial cells (green) in-between. The fluorescent cell types are shown individually and as an overlay image. Scale bar represents 200 µm. (**d**) immunofluorescent picture shows that multiple spheroids can be produced at the same time in a high throughput fashion. Scale bar represents 500 µm. (**B**) Ultrastructural characterization of the cellular morphology of the 3D glomerular co-culture by SEM: (**a**) overview image of the whole 3D spheroid. Scale bar represents 50 µm; (**b**) glomerular endothelial cell with fenestrae-like pores (red boxes). Scale bar represents 2 µm; (**c**) cell–cell interactions of protrusions connecting cells (white arrowheads). Scale bar represents 2 µm; and (**d**) microvilli-like protrusions located on the cell surfaces (white arrowheads). Scale bar represents 2 µm.

The 3D glomerular co-cultures containing all three glomerular cell types formed compact spheroids that were further analyzed by hematoxylin and eosin (HE) staining, multiphoton microscopy and scanning electron microscopy (SEM) (Figure 3A,B). We performed a time-lapse analysis of 3D glomerular co-cultures to investigate self-assembly of the cells. To distinguish between the different cell types of the spheroids in vitro, living glomerular cells were either stained with in vivo dyes or fluorescent reporter cell lines were used. In detail, glomerular endothelial cells stably expressing a tdTomato-Farnesyl reporter were used to label glomerular endothelial cells in red, mesangial cells labeled in green with the eBioscience™ CFSE (ThermoFisher Scientific) and podocytes in blue with the eBioscience™ Cell Proliferation Dye eFluor™ 450. The different glomerular cell types self-arranged in a reproducible manner. Podocytes moved to the center of the spheroid whereas glomerular endothelial cells were located on the outside. Mesangial cells were distributed between podocytes and glomerular endothelial cells (Supplementary Video S1, Figure 3A(b–d)). Multiphoton microscopy confirmed the organization of the cells and displayed the formation of a monolayer with a net-like structure of the GECs encapsulating the spheroid (Figure 3B(a), Supplementary Video S2). SEM analysis of the 3D co-cultured cells showed fenestrae-like pores of the glomerular endothelial cell layer (Figure 3B(b)). Moreover, microvilli-like protrusions on the surface of the cells and interdigitating processes were visible (Figure 3B(c,d)).

### 2.3. Bulk-RNA Sequencing of 3D Glomerular Co-Culture Indicates Gene Expression Similar to In Vivo Condition

**Figure 4:**
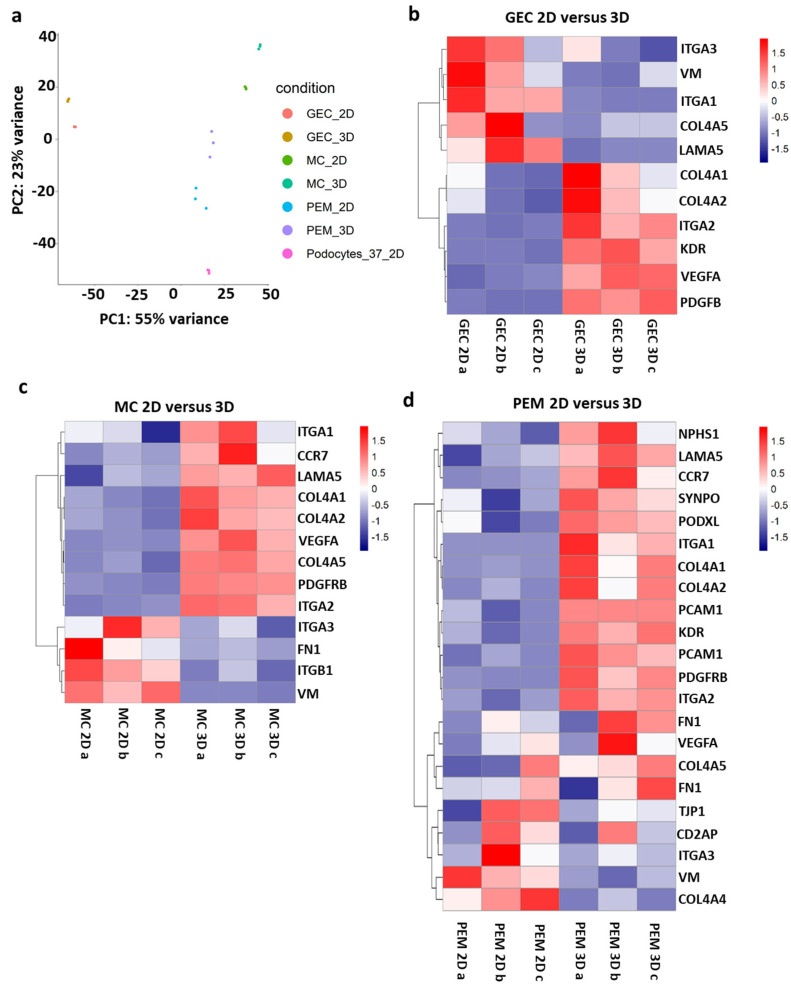
Comparison of transcriptomic data from glomerular monocultures and co-cultures between 2D and 3D culture conditions displays differences regarding cell type-specific marker, extracellular matrix and signaling components: (**a**) principal component analysis of glomerular cell monoculture and co-culture in 2D and 3D. Each condition was analyzed as a biological triplicate. Glomerular endothelial cell (GEC), mesangial cells (MC), co-culture of differentiated conditionally immortalized human podocytes, mesangial cells and glomerular endothelial cells (PEM); and (**b**–**d**) heatmaps representing gene expression of extracellular matrix components, cell type-specific markers, adhesion and signaling molecules of glomerular endothelial cell (GEC) monoculture (**b**), mesangial cell (MC) monoculture (**c**), and glomerular co-culture of podocytes, mesangial cells and glomerular endothelial cells (PEM) (**d**) in 2D and 3D. Each condition was analyzed as a biological triplicate.

To investigate transcriptomic differences based on the culture conditions, bulk-RNA sequencing was performed on 2D and 3D-cultured glomerular mono- and co-cultures. Principal component analysis of the data visualized similarity and variance of the altered culture conditions. While triplicates of each sample and condition clustered together as expected, transcriptomic variation was indicated for 2D and 3D-cultured cells of the same cell type (Figure 4a). These findings were further analyzed by comparison of gene specific expression patterns among the clusters. Especially, expression of cell type-specific markers and extracellular matrix components was compared between the different culture conditions.

In all 3D-cultured monocultures and also in the 3D co-culture, genes encoding for cell type-specific markers, extracellular matrix components, adhesion and paracrine signaling showed enhanced expression. In glomerular endothelial cell monocultures, the typical endothelial specific markers platelet and endothelial cell adhesion molecule 1 (*PECAM1*), the vascular endothelial growth factor A (*VEGFA*), the related kinase insert domain receptor (*KDR*) and the adhesion molecule integrin subunit alpha (*ITGA*) 2 showed enhanced expression when cultured in 3D compared to 2D (Figure 4b). In 3D monoculture of mesangial cells, the expression of the platelet-derived growth factor receptor beta (*PDGFRB*), a mesangial cell marker, was increased. In addition, genes encoding for extracellular matrix components of the glomerular basement membrane, such as collagen type IV alpha (*COL4A*) *1*, *COL4A2*, *COL4A5* and laminin (*LAMA5*), were upregulated in the 3D mesangial cell spheroids. *VEGFA* and *ITGA1* and *ITGA3* were also increased in 3D mesangial culture (Figure 4c). When glomerular endothelial cells, mesangial cells and podocytes were co-cultured in 3D spheroids, gene expression of all cell type-specific markers was likewise increased. More precisely, upregulation of the podocyte-specific markers nephrin (*NPHS1*), synaptopodin (*SYNPO*) and podocalyxin (*PODXL*), the glomerular endothelial cell marker *PECAM1* and the mesangial cell marker *PDGFBR* was detected. Besides, mature extracellular matrix markers, like *COL4A5*, *LAMA5*, *FN1* and the growth factor *VEGFA* and receptor *KDR*, were upregulated compared to 2D culture (Figure 4d). These first results indicate a better physiological environment for the glomerular cells represented by a mature phenotype, extracellular matrix formation and cell–cell interaction and communication.

**Figure 5:**
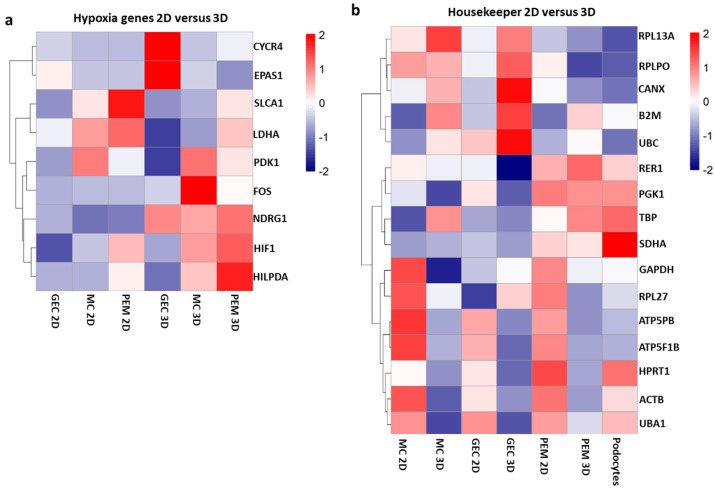
Bulk-RNA sequencing of glomerular monocultures and co-cultures in 2D and 3D exhibits differences in genes involved in hypoxia and housekeeping genes: (**a**) heatmap demonstrating expression of genes involved in hypoxia response in 2D and 3D monoculture of glomerular endothelial cells (GEC) and mesangial cells (MC) as well as 2D and 3D co-cultures of GEC, MC and differentiated, conditionally immortalized human podocytes (PEM); and (**b**) heatmap representing alterations of typically used housekeeping genes in 2D compared to 3D monoculture of GEC and MC as well as 2D and 3D glomerular co-cultures.

Hypoxia-related gene expression was altered in 2D, 3D as well as monoculture or co-culture conditions (Figure 5a). This indicates cellular response to hypoxic conditions. In the 3D co-cultured spheroids, signal transduction via transcription factors was induced by the upregulation of *NDRG1*, *HIF1A* and *HLPDA*.

*PDK1* and *FOS* were upregulated in 3D mesangial cell monoculture compared to 2D condition. In contrast, *EPAS1* and *CYCR4* were upregulated only in 3D glomerular cocultures. These genes play an important role in cell proliferation and differentiation under hypoxic conditions by activating anaerobic energy metabolism and angiogenesis, in order to increase oxygen and nutrient delivery. The different cell types seem to be differently vulnerable for regulating these genes in 3D culture condition. When looking at commonly used housekeeping genes, such as *GAPDH*, *HPRT*, *ACTB*, *UBC* and *SDHA*, we identified significant differences in regulation when comparing 2D to 3D culture conditions (Figure 5b). This finding has very important implications, as normalization techniques with typical housekeepers are probably not suitable when comparing 2D and 3D models, because these genes might be regulated themselves by these culture conditions.

### 2.4. Pathway Analysis of 3D versus 2D Cultured Glomerular Cells

**Figure 6:**
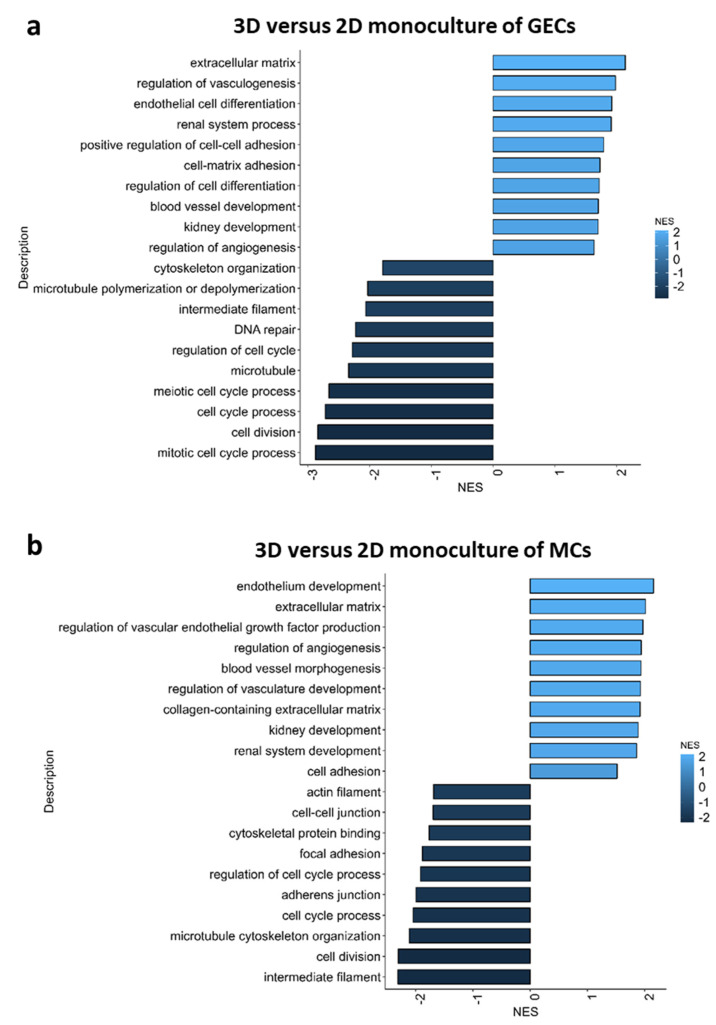

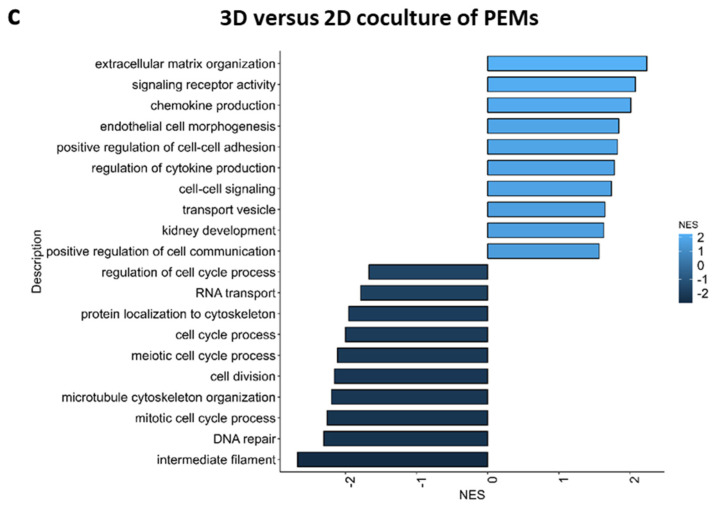
Pathway analysis of bulk-RNA sequencing data from 2D and 3D glomerular cultures. Pathways analysis was performed for monoculture of glomerular endothelial cells (GEC) (**a**), monoculture of mesangial cells (MC) (**b**) and co-culture of podocytes, GECs and MCs (**c**) in 3D or rather 2D. Pathways that were more active in 3D cultures and less active in 2D cultures are colored in light blue. In contrast, pathways that were more active in 2D cultures and less active in 3D cultures were colored in dark blue.

Pathway analysis of bulk-RNA sequencing data revealed that genes involved in extracellular matrix production, regulation of angiogenesis endothelial cell differentiation, cell matrix adhesion, cell–cell adhesion and kidney development were more active in 3D culture conditions. In contrast, gene pathways involved in cytoskeleton formation, DNA repair, cell cycle processes and cell division were less active in 3D cultures (Figure 6a–c).

### 2.5. Validation of Bulk-RNA Sequencing Results by qPCR

In order to validate these findings from bulk-RNA sequencing, we performed real-time qPCR of glomerular endothelial cell, podocyte and mesangial cell cultures as well as glomerular co-cultures of all three cell types, either cultured in 2D and 3D.

**Figure 7:**
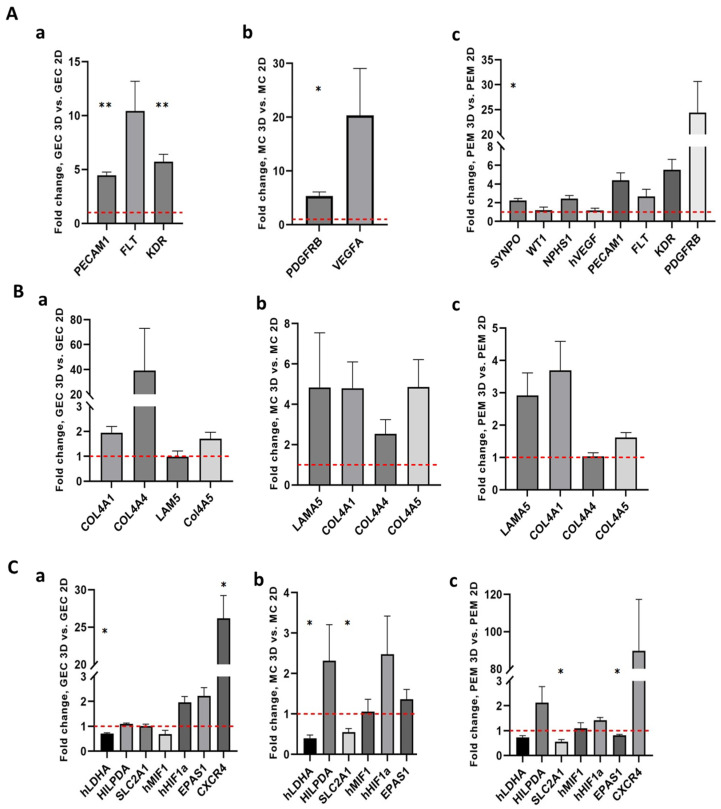
qPCR for cell type-specific markers, extracellular matrix and cellular response to hypoxia of 2D and 3D glomerular cultures. (**A**) qPCR for cell type-specific maker expression and cellular signaling: (**a**) comparison of glomerular endothelial cell (GEC) markers in 3D versus 2D monoculture of GECs; (**b**) Comparison of mRNA expression of mesangial cell (MC) markers in 3D versus 2D monoculture of MCs; (**c**) comparison of mRNA expression of podocyte, MC markers and GEC markers in 3D versus 2D glomerular co-cultures of podocytes, GECs and MCs (PEM). (**B**) qPCR for expression of extracellular matrix: (**a**) comparison of 3D versus 2D monoculture of GECs; (**b**) comparison of mRNA expression of extracellular matrix in 3D versus 2D monoculture of MCs; (**c**) comparison of mRNA expression of extracellular matrix in 3D versus 2D glomerular co-cultures. (**C**) qPCR for expression of hypoxia-related genes: (**a**) comparison between 3D and 2D monoculture of a: GECs and (**b**) mesangial cells; and (**c**) comparison of 3D versus 2D glomerular co-cultures. n = 3 for each experiment. Data are given as mean ± SEM, * *p* < 0.05, ** *p* < 0.01.

Here, the housekeeping genes *HPRT*, *GAPDH* and *ACTB* significantly varied in expression between 2D and 3D culture conditions (Supplementary Figure S4). Thus, they could not be used for normalization. In contrast, we could identify stable expression of RPS18 in 2D and 3D glomerular endothelial cells, podocytes, and mesangial cell cultures (Supplementary Figure S2). Therefore, *RPS18* was used as a housekeeping gene in the following analysis. qPCR confirmed that glomerular endothelial cells expressed significantly more *PECAM 1* and VEGF receptors *FLT* and *KDR* in 3D culture compared to 2D culture conditions (Figure 7A(a)). The mesangial cell marker *PDGFRB* and the growth factor *VEGFA* were also upregulated in 3D mesangial cell spheroids compared to mesangial cell 2D cultures (Figure 7A(b)). Podocyte markers were upregulated in 3D glomerular co-culture compared to 2D glomerular co-culture even though this only reached statistical significance for *SYNPO* (Figure 7A(c)). mRNAs of extracellular matrix, including mature *COL4A4*, *COL4A5* and *LAMA5*, were enriched in 3D conditions compared to 2D cultures confirming results from bulk-RNA sequencing (Figure 7B(a–c)).

### 2.6. Comparison of Protein Levels between 2D and 3D-Cultured Glomerular Cells

**Figure 8:**
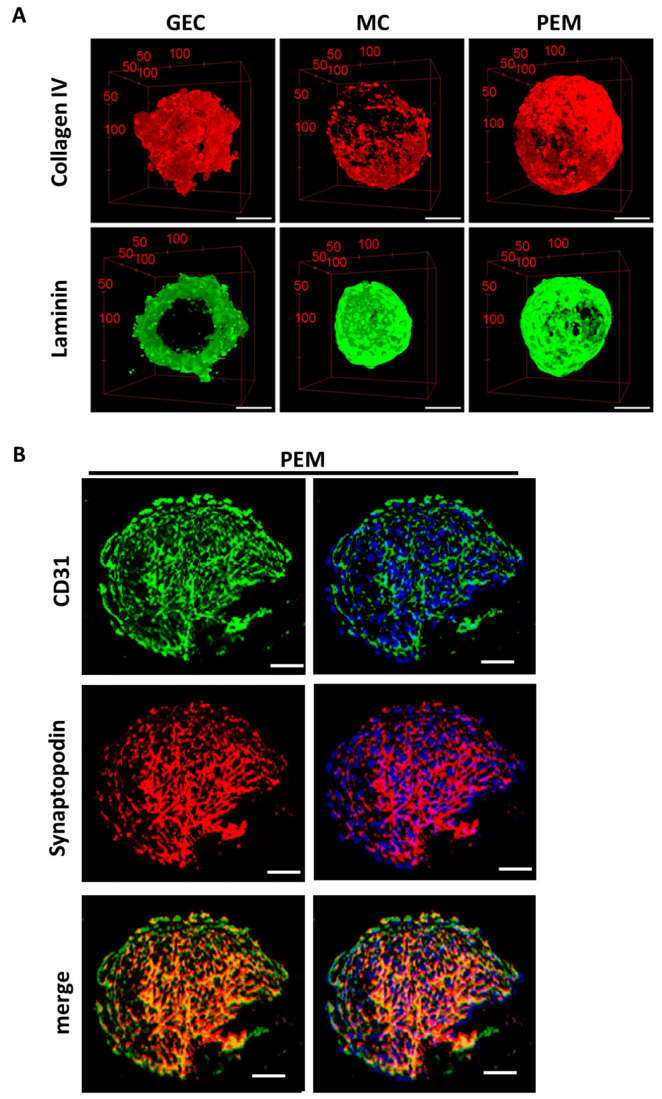
Immunofluorescence staining showing the production of extracellular matrix and the localization of podocyte-specific and glomerular endothelial cell-specific marker proteins in 3D glomerular co-cultures. (**A**) 3D reconstructions of multiphoton microscopy image-stacks of glomerular spheroids showing collagen IV (red) and laminin (green) expression in glomerular endothelial cell (GEC) monoculture spheroids, mesangial cell (MC) monoculture spheroids and glomerular co-culture (PEM). Scale bar represents 50 µm. (**B**) Confocal microscopy images of glomerular co-culture spheroids (PEM) demonstrating CD31 (green), synaptopodin (red) and merged images. Nuclear staining is shown in blue. Scale bar represents 50 µm.

Multiphoton microscopy imaging of stained 3D glomerular spheroids displayed production of the typical glomerular extracellular matrix components collagen IV and laminin. These proteins were evenly distributed throughout the whole spheroid. 3D glomerular endothelial cell monocultures produced more collagen IV, whereas mesangial cell spheroids produced more laminin (Figure 8A). In 3D glomerular co-cultures, the endothelial cell marker CD31 was also located at the periphery of spheroids, while the podocyte marker synaptopodin was mostly found in the center of the spheroids. This is in line with the structural organization of the cells that could be observed before (Figure 8B).

### 2.7. Cell–Cell Communication within the 3D Glomerular Spheroids

**Figure 9:**
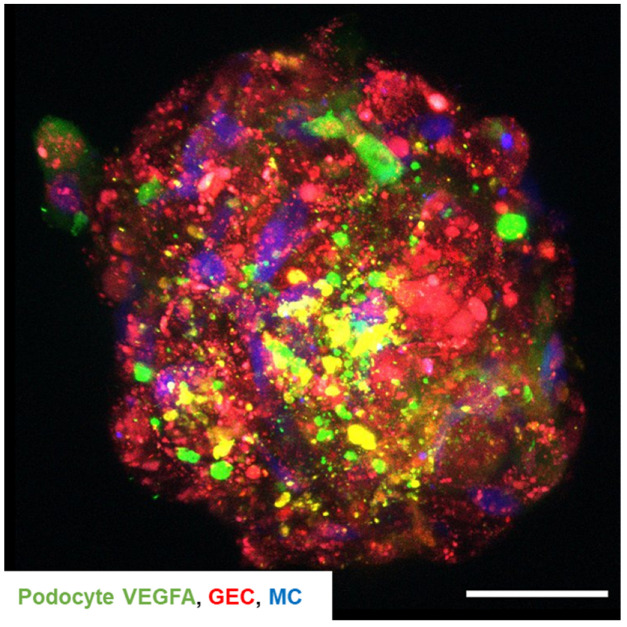
Podocyte-derived VEGFA is transported to glomerular endothelial cells in 3D glomerular co-cultures. Confocal microscopy of 3D glomerular co-culture displays green fluorescent podocyte-derived VEGFA, mesangial cells labeled in blue with eBioscience™ Cell Proliferation Dye eFluor™ 450 and red fluorescent tdTomato-Farnesyl glomerular endothelial cell reporter cells. Podocytes were electroporated with a plasmid carrying human VEGFA sequence coupled with the GFP sequence, resulting in the expression of green, fluorescent VEGFA prior to co-culture. Scale bar 50 µm.

Next, we investigated cell–cell communication within the 3D glomerular co-culture of glomerular endothelial cells, mesangial cells, and podocytes. Therefore, we electroporated conditionally immortalized human podocytes with a plasmid encoding green fluorescent VEGFA, co-cultured all three glomerular cell types and subsequently live-tracked VEGFA localization. Here, a colocalization of the green-fluorescent podocyte-derived VEGFA was detected with the tdTomato-Farnesyl glomerular endothelial cells (Figure 9). This finding demonstrated that paracrine signaling is possible within the 3D glomerular co-culture.

## 3. Discussion

Currently, 2D monocultures of glomerular cell types are widely used in basic renal research. However, these models lack 3D context, cell–cell interaction and paracrine signaling between cell types of the glomerulus. Furthermore, podocyte cell lines cultured in 2D monocultures express less than 5% of synaptopodin and nephrin proteins as well as the *NPHS1* and *NPHS2* mRNAs of glomerular levels. Moreover, most podocyte cell lines do not express nephrin [32,33].

Thus, cell culture models that better mimic glomerular conditions ex vivo are needed. In general, 3D models can be classified into scaffold-based or non-scaffold-based technologies, for which the latter rely on self-aggregation of the cells in specialized environments [30]. Kidney organoids have the potential for 3D microtissue modelling ex vivo. Here, the cell culture model is based on pluripotent stem cell differentiation in a 3D environment with potential for the development of several cell types and structural components. Despite the promising features of organoids, they also display a variety of limitations, including limited maturation. This is why they are often used in developmental models. Atypical physiology, difficulties to reproduce at scale, considerable variability in organoid formation and complex and expensive protocols for their generation are drawbacks of organoids [10,34,35,36]. In contrast, 3D spheroids are produced with terminally differentiated cells, enabling the investigation of cell–cell interaction and communication ex vivo in an easy and fast way. The glomerular spheroids described here demonstrated highly reproducible self-assembly, survived more than two weeks in the cultures, and presented a mature phenotype due to the production of a mature extracellular matrix and expression of cell type-specific glomerular markers. Even the generation of cellular signaling components, such as the growth factors VEGFA and its receptors could be demonstrated. Moreover, agarose molds with 313 micro-wells enabled high throughput generation and analysis of the glomerular spheroids.

Hybrid 3D spheroids composed of mouse podocytes, mesenchymal stem cells, and HUVECS were generated before [31]. The spheroids generated here are composed of mature and differentiated human podocytes, human glomerular endothelial cells, and human mesangial cells. Therefore, these spheroids much better resemble the human glomerular environment ex vivo. Tuffin et al. [30] used a scaffold of methylcellulose hydrogel for the generation of spheroids, whereas our spheroids formed without any scaffolds, purely by gravity. Another glomerular spheroid model called ‘GlomSphere’ has been published before, but that model lacked glomerular mesangial cells, an important component of the glomerulus regarding structure and cell communication. Moreover, comparative characterization of the 3D models was not performed in such detail before [30].

Here, all three relevant human glomerular cell types were integrated in 3D co-cultures using self-made agarose micro-wells. With the help of fluorescent staining of living cells and subsequent time-lapse analysis and SEM imaging, it was possible to track aggregation and organization of the cells without an additional scaffold inside the spheroids. The glomerular spheroids described here are highly reproducible, easy and fast to generate, and suitable for large-scale screening experiments. Interestingly, 3D monoculture of the podocytes was not possible as the cells did not form stable 3D complexes and showed increased cell death. In contrast, culturing podocytes in the presence of glomerular endothelial and mesangial cells in 3D resulted in stable spheroid formation, and better cell survival and maturation. This indicates that podocytes benefit from co-culturing with these glomerular cell types in 3D. They might also be dependent on extracellular matrix proteins produced by other cells in 3D [31,37].

In the 3D glomerular co-cultures, a specific pattern of self-organization of the cells with podocytes in the center, endothelial cells encapsulating the spheroids, and mesangial cells in between as support cells, was apparent, and different to the structural organization of the glomerulus in vivo. However, this can most likely be explained by the fact that podocytes do not proliferate due to their terminally differentiated state, while endothelial cells still divide and have more space at outer parts of the spheroid. Mesangial cells are in contact with podocytes as well as glomerular endothelial cells and, therefore, were found to be distributed throughout the spheroid.

Furthermore, we compared 3D co-cultures to 3D monocultures as well as 2D co-cultures and 2D monocultures of glomerular cells by bulk-RNA sequencing. Genes involved in cell differentiation, extracellular matrix production, regulation of angiogenesis, cell-matrix adhesion, cell–cell adhesion, and kidney development were upregulated in 3D glomerular cultures compared to 2D cultures. It seems logical that cells must remodel cell adhesion and cellular matrix receptor interaction to detach from the underlying cell culture dish and grow in 3D manner. Cell differentiation was most likely increased due to paracrine signaling from other cell types. This also corresponded to the upregulation of podocyte-, endothelial and mesangial cell-specific marker expression in 3D cultures. Genes involved in cytoskeleton formation, DNA repair, cell cycle processes and cell division were downregulated in 3D cultures. This suggests that there might be less cell damage in 3D and again points to a more differentiated state of the cells in 3D culture conditions.

Glomerular growth factors such as *VEGFA* and *PDGFB*, their receptors *KDR*, *FLT1* and *PDGFBR* were upregulated in 3D glomerular co-cultures. Paracrine PDGF-B/PDGF-R beta signaling controls mesangial cell development in kidney glomeruli [38]. Intraglomerular crosstalk between podocytes and glomerular endothelial cells through VEGF-A is important for glomerular functions [39]. Mature extracellular matrix proteins such as *COL4A5* and *LAMA5* were also higher expressed in 3D glomerular cocultures compared to 2D monocultures. These collagen and laminin types are indeed the once expressed in the glomerular basement membrane. GBMs of nephrons contain laminin α1β1γ1 heterotrimers, whereas those of maturing glomeruli contain laminin α5β2γ1 and laminin α5β1γ1 [40]. Similarly, the most immature GBMs of early nephrons contain networks of collagen α1α2α1(IV), and those of fully mature nephrons contain collagen α3α4α5(IV) [32,41]. Immunoelectron microscopy and metanephric grafting experiments showed that endothelial cells and podocytes both secrete collagen α1α2α1(IV), but collagen α3α4α5(IV) originates solely from podocytes [42]. Fully mature nephrons contain collagen α3α4α5(IV) and laminin5 [32,41]. Podocyte-specific markers such as *NPHS1*, *SYNPO* and *PODXL* were enriched in 3D glomerular cocultures. The role of podocyte markers is especially noticeable when it comes to podocytopathies, where these genes are mutated or decreased [43] and as has already been mentioned above, the lack of expression of podocyte markers is often a problem in 2D cultured immortalized podocytes.

These results indicate that glomerular cells not only need paracrine interaction but also 3D culture conditions to better recreate the in vivo situation. Therefore, it is beneficial to integrate all three glomerular cell types in a 3D co-culture model to provide earlier reported cell–cell communication between podocytes and mesangial cells, podocytes and endothelial cells, and also mesangial cells and endothelial cells [44]. It was shown before that co-culture of podocytes and endothelial cells resulted in an altered composition and organization of extracellular matrix compared to a monoculture, which was thought to be regulated by intercellular crosstalk [45]. However, those experiments did not consider mesangial cells and 3D cell culture conditions. Here, it was shown that not only co-culture but also 3D cell culture influences the phenotype of glomerular cells. Genes involved in hypoxia were altered depending on their cultivation in 2D or 3D. Especially, *NDRG1* was upregulated in 3D cultures of all cell types compared to 2D. NDRG1 was previously reported to be an important marker responsible for cell trafficking under hypoxic conditions [46,47].

Commonly used housekeeping genes for qPCR analysis normalization include *ACTB*, *GAPDH*, UBC, *HPRT*, or *SDHA*. However, many of these genes showed inacceptable variability in expression [48,49,50]. We analyzed 16 different housekeeping genes and found astonishing differences in the regulation between the 3D and 2D cultivation in all these genes. For instance, we identified *RPS18* as being stably expressed between 2D and 3D cultures in glomerular endothelial cells, mesangial cells, and glomerular co-cultures including podocytes.

Bulk-RNA sequencing results were confirmed by qPCR and showed an increase in podocyte and glomerular endothelial cell-specific marker expression in 3D glomerular co-cultures. Interestingly, hypoxia genes were altered in 3D cultures with some genes being upregulated and others being downregulated. Genes involved in hypoxia, redox signaling and cytoskeleton assembly have also been reported to be upregulated in 3D culture of non-glomerular cells [27,28,29]. Furthermore, an oxygen and nutrition gradient from the outer to the inner part of spheroids has been simulated before [20,51]. Here, incorporation of, for example, fibers to increase nutrition supply inside the spheroid could be improved in future experiments [52].

Transport and colocalization of podocyte VEGFA to glomerular endothelial cells was visualized with the help of fluorescently labeled mesangial cells, tdTomato-Farnesyl glomerular endothelial cell reporter cell line and the insertion of the green fluorescent VEGFA plasmid into podocytes. The experimental goal was not to simulate in vivo VEGFA crosstalk, as the typical glomerular basement membrane is lacking in this model. Instead, it showed that the improved maturation and altered expression profile of 3D glomerular co-culture was indeed due to a cell–cell communication between the different cell types. VEGFA was secreted from podocytes and translocated to glomerular endothelial cells that themselves do not express VEGFA.

In summary, our findings have important relevance for the interpretation of data from glomerular culture models. We showed the increased expression of cell type-specific markers, extracellular matrix proteins and genes involved in cell–cell communication due to co-culture or the cultivation in 3D. Finally, yet importantly, this analysis demonstrated that normalization techniques involving many typical housekeepers are not suitable when comparing 2D and 3D glomerular cell culture models, as gene regulation is altered. Therefore, choosing genes wisely for further data normalization is extremely important.

The distinct self-organization of cells within the spheroids was highly reproducible without any need for a scaffold and proved to be beneficial for podocyte survival and maturation. Changes in cell type-specific marker expression could be validated from independent bulk-RNA sequencing with qPCR analysis. The 3D glomerular co-culture model generated here is suitable for further ex vivo studies of intercell–cell communication and makes an important contribution for studies in disease modelling or drug screening.

## 4. Materials and Methods

### 4.1. Cell Culture

Conditionally immortalized human podocytes (kindly provided by Moin Saleem, Children’s and Renal Unit and Bristol Renal, University of Bristol) were proliferated under permissive conditions at 33 °C. When cultivated at 37 °C, the SV40 T-antigen was inactivated for terminal cell differentiation. Human podocytes were cultured in RPMI Medium 1640 (Gibco, Thermo Fisher Scientific, Waltham, MA, USA) supplemented with 10% heat-inactivated fetal bovine serum (FBS, PAN-Biotech, Aidenbach, Germany), 1% penicillin-streptomycin (Sigma-Aldrich, Merck, St. Louis, MO, USA), and 0.1% insulin-transferrin-selenium (ThermoFisher Scientific, Waltham, MA, USA).

Primary human glomerular mesangial cells (MC, ACBRI 127) and primary human glomerular microvascular endothelial cells (GEC, ACBRI 128) were purchased from Cell Systems, Kirkland, WA, USA, human mesangial cells were cultured in RPMI Medium 1640 supplemented with 10% heat-inactivated FBS and 1% penicillin-streptomycin. Primary human glomerular endothelial cells were maintained in commercially available endothelial cell media (VascuLife^®^ VEGF-Mv Medium, LifeLine^®^ Cell Technology, ThermoFisher Scientific, Waltham, MA, USA) containing 5 ng/mL rhFGF basic, 50 µg/mL ascorbic acid, 1 µg/mL hydrocortisone hemisuccinate, 10 mM L-glutamine, 15 ng/mL rhIGF-1, 5 ng/mL rhEGF, 5 ng/mL rhVEGF, 0.75 U/mL heparin sulfate, 5% fetal bovine serum, 30 mg/mL gentamycin, and 15 µg/mL amphotericin B (all supplements, LifeLine^®^ Cell Technology, ThermoFisher Scientific, Waltham, MA, USA).

### 4.2. Glomerular Endothelial Cell Reporter Cell Line

The human glomerular endothelial cell reporter cell line, stably expressing the tdTomato fluorescent protein coupled to a plasma membrane-targeting farnesylation-sequence, was generated by lentiviral transduction. The cDNA of the farnesylated tdTomato protein was amplified from tdTomato-Farnesyl-5 (#58092, Addgene, Watertown, MA, USA) using a 5′-primer encoding a BamHI site and a 3′-primer encoding a NotI site and cloned into pLVX-AcGFP-N1 (#632154, Clontech Laboratories Inc.; Mountain View, CA, USA) to replace the AcGFP cDNA. Using the the NucleoBond Xtra Maxi Kit (Macherey–Nagel, Hoerdt, France; #740414.50), the reporter plasmid DNA was purified before its sequence was verified by custom DNA sequencing (Eurofins Genomics Germany GmbH, Ebersberg, Germany). To generate stable tdTomato-Farnesyl-expressing hGEC cells, LentiX 293T cells (Takara Bio Europe SAS, Saint-Germain-en-Laye, France) were first transfected with the transfer plasmid pLVX-tdTomato-Farnesyl-N1, the packaging plasmid psPAX2 (#2260, Addgene, Watertown, MA, USA) and the envelope plasmid pCMV-VSV-G (#8454, Addgene, Watertown, MA, USA), using Lipofectamine 2000 reagent (Invitrogen-Thermo Fisher Scientific, MA, USA). Plasmid map of lentiviral transfer vector that carries the sequence for a tdTomato protein with an additional farnesylation signal is given in Supplementary Figure S3. After 48 h, the lentivirus-containing supernatant was harvested and pre-cleaned by quick centrifugation (500× *g*, 10 min). The virus-containing medium was concentrated (10×) using LentiX-concentrator (Takara Bio Europe SAS, Saint-Germain-en-Laye, France) and subsequently used to transduce glomerular endothelial cells which were seeded at a density of 2 × 10^4^ cells per 9.6 cm^2^ on top of the pre-seeded lentivirus (reverse transduction). To further enhance its efficiency, the reaction was supplemented with 10 µg/mL polybrene (Sigma-Aldrich). Successfully transduced cells were further selected with puromycin (1 µg mL^−1^) (Gibco™-Thermo Fisher Scientific, Waltham, MA, USA).

### 4.3. 3D Cell Culture

Agarose micro-wells for spheroid formation were generated using 3D-printed negative templates with 313 peaks (Supplementary Figure S4). These were kindly produced by the group of Dr. Ahmad from the Department for Functional Materials in Medicine and Dentistry, Würzburg University, Germany. These molds were covered with 2.5% agarose solution. After polymerization, agarose wells, containing 313 micro-wells with a diameter of 500 µm each, were cut out and sterilized using UV light. The cell suspension was added into the agarose molds allowing cells to aggregate into 313 spheroids by gravity. Spheroid cultures were maintained for four days unless otherwise stated. For the 3D monocultures, 5 × 10^3^ cells per spheroid were used. For the 3D co-cultures, 3 × 10^3^ human podocytes, 0.6 × 10^3^ glomerular endothelial cells, and 0.6 × 10^3^ mesangial cells were mixed per spheroid. This ratio was applied because of the inability of terminally differentiated podocytes to proliferate. The medium was exchanged every second day.

### 4.4. Live/Dead Assay

With the LIVE/DEAD™ Cell Imaging Kit (ThermoFisher Scientific, Waltham, MA, USA), living cells can be distinguished based on intracellular esterase activity determined by the enzymatic conversion of the non-fluorescent cell-permeable calcein AM to the fluorescent calcein which is well-conserved in living cells. The red BOBO-3 Iodide is impermeable for living cells and therefore only enters dead cells with damaged membranes. The kit was used according to the manufacturer’s instructions. For the dead-cell control, spheroids were incubated overnight in 100 μL 4% PFA at 37 °C. The Acquifer imaging machine was used for the analysis.

### 4.5. RNA Isolation

The total RNA of 2D and 3D samples was isolated using the ReliaPrep™ RNA Cell Miniprep System (Promega, Madison, WI, USA) according to the manufacturer’s protocol. Additionally, lysed samples were homogenized using QIAshredder (Qiagen, Venlo, The Netherlands) before RNA isolation. For reverse transcription into cDNA, 500 ng of RNA was mixed with a master mix containing 5× RT-buffer, 10 mM dNTPs, random hexamer primers, reverse transcriptase 1 (Promega, Madison, WI, USA), and RiboLock (ThermoFisher Scientific, Waltham, MA, USA). For qPCR, analysis was performed in triplicates using SYBR green (ThermoFisher Scientific, Waltham, MA, USA) with the StepOnePlus Real-Time PCR System (ThermoFisher Scientific, Waltham, MA, USA). Primer sequences are given in Table 1.

### 4.6. Bulk-RNA Sequencing

Bulk-RNA sequencing was performed for human glomerular endothelial cells, conditionally immortalized human podocytes and human mesangial cells cultured in 2D and 3D monocultures or co-cultures. RNA quality was checked using a bioanalyzer, and samples with an RNA Integrity Number > 8 were sequenced. For library preparation, a protocol of Novogene was utilized. Samples were sequenced with a fragment size of 150 bp and around 30 million reads using Illumina Novaseq 6000 (Illumina, San Diego, CA, USA). Quality check was performed using FastQC (v0.11.8). Subsequently, the STAR alignment software (v2.6.1c) was operated to map the reads to the human reference genome (hg38). A table of counts was generated using the feature-counts software (v1.6.1), and raw reads were sorted normalized and visualized using R-Studio (v4.1.1). Reads from mitochondrial DNA were excluded, and only genes with a minimum of ten counts were analyzed. Data transformation and exploration was performed using DESeq2 package (v1.32.0). Normalization of the gene counts was implemented using variance-stabilized transform. Subsequently, the principal component analysis (PCA) was accomplished using the DESeq2 package with the plotPCA function and visualized with ggplot2 (v3.3.6). Differentially expressed genes (DEGs) were analyzed with a lfcshrink approach, and the Benjamini–Hochberg method within DESeq2 was applied for generation of adjusted *p*-values. DEGs with a false discovery rate (FDR) < 0.05 and log_2_(fold change) ≥ log_2_(0.5) or ≤ –log_2_(0.5) were specified as upregulated and downregulated, respectively. For addition of the gene ID, HUGO Gene Nomenclature Committee (HGNC) and Entrez gene ID biomaRT (v2.48.3) was the tool of choice. Raw reads were normalized by transcripts per million (TPM) using R-Studio and gene annotations were added using biomaRt (v2.50.3) and plotted using the heatmap package (v1.0.12). Gene-set-enrichment analysis (GSEA) was performed according to Subramanian, A. et al. [53]. Genes showing a significant difference of FDR < 0.05 corresponding to DESeq2 and fold differences were used for the Entrez gene ID annotation. Log_2_(fold change) values were taken as a ranking score. Functional enrichment analysis of the gene ontology gene sets was analyzed regarding biological processes, molecular function, and cellular component via clusterProfiler (v4.2.2) with the gseGO function and minGSSize of 5 and maxGSSize of 800 parameters [54]. Annotation of gene names HGNC to results was applied using org.Hs.eg.db (v3.14.0) (Bioconductor, Marc Carlson (2021; https://www.bioconductor.org/). Plotting of the GSEA results was conducted using ggplot2 (v3.3.6).

### 4.7. Immunocytochemistry-Staining

Samples were washed with 1× PBS and fixed with 4% PFA (Roth, Karlsruhe, Germany) for 15 min. To permeabilize and block unspecific binding sites, samples were incubated with 1× PBS containing 10% normal goat serum (NGS, Abcam, Cambridge, UK), 1% bovine serum albumin (BSA, Karlsruhe, Germany), 0.5% Triton X–100 (Merck, Darmstadt, Germany) for 1 h at room temperature (RT). Subsequently, samples were labeled with primary antibodies (Table 2) diluted in 1× PBS containing 3% NGS and 1% BSA overnight at 4 °C. The next day, samples were washed three times with 1× PBS with subsequent incubation in the dark and at RT for 1 h with secondary antibodies (A-31634, goat anti-rabbit 647 (Thermo Fisher Scientific, Waltham, MA, USA), goat anti-mouse 488 (A-21244, Thermo Fisher Scientific, Waltham, MA, USA) or donkey anti-rabbit (A-31572, Thermo Fisher Scientific, Waltham, MA, USA). These were diluted in the same buffer as the primary antibodies. Next, nuclei were stained with Hoechst (1:200 dilution in 1× PBS for 10 min in the dark). After washing with 1× PBS, cells that were cultured on coverslips were mounted with a drop of Fluoromount-G™ Mounting Medium (Invitrogen, Thermo Fisher Scientific, Waltham, MA, USA) on a microscope glass slide. Spheroids were placed on a cover slide, covered with mounting medium, and immediately imaged using a laser scanning confocal microscope.

### 4.8. Labeling Living Cells with In Vivo Dyes

Cell suspension of conditionally immortalized human podocytes was stained with 2 µM of the eBioscience™ Cell Proliferation Dye eFluor™ 450 (Invitrogen, ThermoFisher Scientific, Waltham, MA, USA), and mesangial cells were labeled with 1 µM of the eBioscience™ CFSE (Invitrogen, ThermoFisher Scientific, Waltham, MA, USA) according to the manufacturer’s instructions. Cells were washed with 1× PBS to remove serum components of the media and subsequently centrifuged. Then, cells were resuspended in 1× PBS containing the desired final concentration of the dyes and incubated for 10 min at 37 °C (for e450) or RT (for CFSE) in the dark. After stopping the labeling reaction by adding 5× the volume of the staining solution with cold media for 5 min on ice, cells were washed three times with media. The time-lapse experiments were performed using the Aquifer imaging machine immediately after staining. Live images were taken every 30 min for 24 h.

### 4.9. Multiphoton Microscopy

Multiphoton imaging was performed using multifocal multiphoton microscope (TrimScope II, LaVision BioTec, Bielefeld, Germany) in combination with a mode-locked femtosecond-pulsed Ti:Sa laser (Chameleon Vision II, Coherent, Santa Clara, CA, USA) at a pulse frequency of 80 MHz and an average input power of 58–510 mW, depending on sample size and staining type. The laser was focused into the sample through a 25× water immersion objective with a numerical aperture of 0.95 (Leica HC Fluotar L 25×/0.95 W VISIR, Leica Microsystems, Wetzlar, Germany) and tuned to an excitation wavelength of 810 nm. The backward directed fluorescence signal was detected with three ultrasensitive photomultiplier tubes (H 7422-40 LV 5M, Hamamatsu Photonics, Herrsching, Germany) and spectrally separated with varying sets of dichroic mirrors/longpass filters (Chroma ET-series, Chroma Technology Corporation, Bellow falls, VT, USA) and bandpass filters (Chroma T-series, Chroma Technology Corporation, Bellow falls, VT, USA), given in Table 3. To allow for the extraction of three-dimensional morphological and structural information, the recording of XYZ volumetric image stacks was used as the imaging methodology of choice. The imaging parameters were set to a lateral pixel size of 0.4 µm, a line-scanning frequency of 1000 Hz and a step size in axial direction of 1 µm for the glomerular co-culture and 0.4 µm for the single-culture samples. These settings resulted in a pixel dwell time of 0.7 µs and a physical voxel size of 0.4 × 0.4 × 0.4/1 µm. Image analysis and 3D-reconstruction of the image data were done with FIJI/ImageJ [55].

### 4.10. Design of Human VEGFA-GFP Plasmid and Transfection of Podocytes

A human VEGFA sequence coupled with a GFP sequence was cloned into a mammalian gene-expression vector (pLV[Exp]-Puro-CMV>3xFLAG/ORF_Stuffer, #VB900138-3673 twt, Vectorbuilder, Chicago, IL, USA). After transformation and expansion in *E. coli*, the plasmid was purified using the QIAprep Spin Miniprep Kit (QIAGEN). Undifferentiated conditionally immortalized human podocytes were transfected with 5 µg VEGFA plasmid using the ProGenetor II electroporator (Hoefer, Holliston, MA, USA) with one pulse at 280 V and 1200 µF. Subsequently, after electroporation, cells were transferred to 37 °C for differentiation. After two days, VEGF-GFP-expressing podocytes were mixed with red fluorescent tdTomato-Farnesyl glomerular endothelial cell reporter cells and e450 stained MC, as described before (Labeling living cells with in vivo dyes). After four days of coculturing, spheroids were fixed with 4% PFA and imaged using a confocal microscope.

### 4.11. Confocal Microscopy

Confocal imaging was performed using a Leica DMI6000 inverted confocal microscope (Leica Microsystems, Wetzlar, Germany) in combination with the LAS X Software (v2.0.1.14392). Samples were imaged using the following objectives: HC PL APO 20×/0.50 DRY, HC PL APO 40×/1,40 OIL CS2, HC PL APO 63×/1,40 OIL CS2. Image processing was done with FIJI/ ImageJ-win32, Version v1.53u [55].

### 4.12. Scanning Electron Microscopy

Spheroids were fixed for 1 h with 2% glutaraldehyde in 0.1 M Sodium cacodylate buffer, pH 7.4, rinsed three times for 15 min in 0.1 M sodium cacodylate buffer, pH 7.4 and post-fixed 1 h in 1% Osmium tetroxide in water following another three rinses in water. Dehydration was performed with an increasing series of ethanol (50% for 5 min, 70% for 5 min, 70% for 5 min, 95% for 5 min, two times 100% for 10 min). After dehydration, the cells were dried in a critical point dryer. Electron micrographs were taken with the Zeiss Auriga FIB/FE-SEM (AURIGA TM^®^ Crossbeam Workstation, Carl Zeiss NTS GmbH, Oberkochen, Germany) microscope.

### 4.13. Statistics

All data are expressed as mean ± SEM, where SEM refers to standard error of the mean. The distribution normality was tested using a Shapiro–Wilk test. For comparison of mean values between two groups, a one sample *t*-test (compared to 1) was used. One sample Wilcoxon test (compared to 1) was applied for comparison of data with non-parametric dispersion. Statistical significance was evaluated using GraphPad Prism. The experimental findings were considered statistically significant if *p* < 0.05.

## Figures and Tables

**Table 1:** Primer sequences for qPCR.

Gene	Encoding for	Sequence (5′ -> 3′)
*hHPRT*	Hypoxanthine phosphoribosyltransferase 1	GACCAGTCAACAGGGGACAT AACACTTCGTGGGGTCCTTTTC
*hGAPDH*	Glyceraldehyde-3-phosphate dehydrogenase	CAAGATCATCAGCAATGCCTCC ATGATGTTCTGGAGAGCCCC
*hRPS18*	RPS18 rRNA	GTTGATTAAGTCCCTGCCCTTTGT CGATCCGAGGGCCTCACTA
*hWT1*	Wilms tumor protein	TTATTGCAGCCTGGGTAAGC TCAGAGGCATTCAGGATGTG
*hSYNPO*	Synaptopodin	TAAGCAACC TTCTGGGCTAAAGCTAAC
*hNPHS1*	Nephrin	TCACATCTCCATGTCCAACC GGGCGGGATATTTTACGTTC
*hVEGFA*	Vascular endothelial growth factor A	CAACAAATGTGAATGCAGACCAAA CCCTTTCCCTTTCCTCGAACT
*hPECAM1*	CD31	TGGAAAGCAGATACTCTAGAACGG GGGATGTGCATCTGGCCTT
*hKDR*	Kinase insert domain receptor, VEGFR2	TGGTTGTGTATGTCCCACCC GGAGGAATGGCATAGACCGT
*hFLT1*	Fms-related receptor tyrosine kinase 1, VEGFR1	TGTGAAAATGCTGAAAGAGGGGG AGATGGTGGCCAATGTGGGT
*hPDGFB*	Platelet derived growth factor subunit B	GTTTATCATGGGCCTCGGGGA TCATCAAAGGAGCGGATCGAG
*hLAMA5*	Laminin 5	GGAGAACGGAGAGATCGTGG CAGCGGCGAGTAGGAGAAAT
*hCOL4A1*	Collagen IVα1	GTCTGGCTGCTGCTGCT CACAGCCACCCTTCGCA
*hCOL4A4*	Collagen 4α4	GCTTCTTGCACTCACAACGG GCTCCTGTAACAGCCAACCA
*hCOL4A5*	Collagen 4α5	GGCCCCAAGGTCCTCCT TCCACTGGGTCCTTTCATGC
*LDHA1*	Lactat dehydrogenase A	CATAGCTGTTCCACTTAAGGCCC TGCCATATTGGACTTGGAACC
*hHIG2*	Hypoxia-inducible gene 2	AACACATGCTTCATGGCTGAAAGG TCTGCGCTGGTGCTTAGTAACC
*hGLUT 1*	Glucose transporter 1	ACCATTGGCTCCGGTATCG GCTCGCTCCACCACAAACA
*hMIF*	Macrophage migration inhibitory factor	CGGGTTCCTCTCCGAGCT CCGATCTTGCCGATGCTGT
*hHIF1a*	Hypoxia-Inducible Factor 1-Alpha	GAAGTGGCAACTGATGAGCA GCGCGAACGACAAGAAA
*hHIF2a*	Hypoxia-Inducible Factor 2-Alpha	GTCTGAACGTCTCAAAGGGC CTTCTCCTTCCTCCTCTCCG
*hACTB*	beta actin	ACCGAGCGTGGCTACAGCTTCACC AGCACCCGTGGCCATCTCTTTCTCG

**Table 2:** Primary antibodies.

Antigen (Clone)	Host	Company, Cat. No.	Dilution
Collagen IV	rabbit	Abcam, Cambridge, UK, ab6586	1:100
Laminin 1/2	rabbit	Novus Bio, Littleton, CO, USA, NB300-144SS	1:100
CD31	mouse	ThermoFisher Scientific, Waltham, MA, USA, MA5-13188	1:100
Synaptopodin	rabbit	Proteintech, Rosemont, IL, USA, 21064-1-AP	1:100

**Table 3:** Optical filter setup of the multiphoton microscope, considering different sample types and fluorescence staining.

		PEM (Figure 3)	PEM (Figure 8)	GEC (Figure 8)	MC (Figure 8)
	Dichroic mirrors	495 nm	560 nm	560 nm	560 nm
		560 nm	n.a.	n.a.	n.a.
Fluorophores	tdTomato	572/35 nm	n.a.	n.a.	n.a.
	CFSE	525/50 nm	n.a.	n.a.	n.a.
	E450	450/30 nm	n.a.	n.a.	n.a.
	Alexa Fluor 647	n.a.	675/67 nm	675/67 nm	675/67 nm

## Data Availability

The main data supporting the findings of this study are available within the paper. Bulk sequencing data will be made available upon request. Further information and requests for resources and reagents should be directed to the corresponding author.

## Supplementary Materials

The supporting information can be downloaded at: https://www.mdpi.com/article/10.3390/ijms241210384/s1.
