# Supplementary material for: Partner, Neighbor, Housekeeper and Dimension: 3D versus 2D Glomerular Co-Cultures Reveal Drawbacks of Currently Used Cell Culture Models
Source: Int J Mol Sci. 2023 Jun 20;24(12):10384. doi: 10.3390/ijms241210384 (PMC10299010; doi:10.3390/ijms241210384)
Supplement: Supplementary file 1 [file ijms-24-10384-s001.zip › Supplementary data.pdf]

Supplementary data

Supplementary Video S1: *Representative video of glomerular co-culture in 3D over a time course of four days.* Podocytes are labeled in blue (eBioscience™ Cell Proliferation Dye eFluor™ 450), glomerular endothelial cells are labeled in red (tdTomato-Farnesyl-reporter line) and mesangial cells are labeled in green (eBioscience™ CFSE).

Supplementary Video S2: *3D reconstruction and animation of multiphoton images of glomerular co-culture in 3D.* Podocytes are labeled in blue, glomerular endothelial cells are labeled in magenta and mesangial cells are labeled in green.

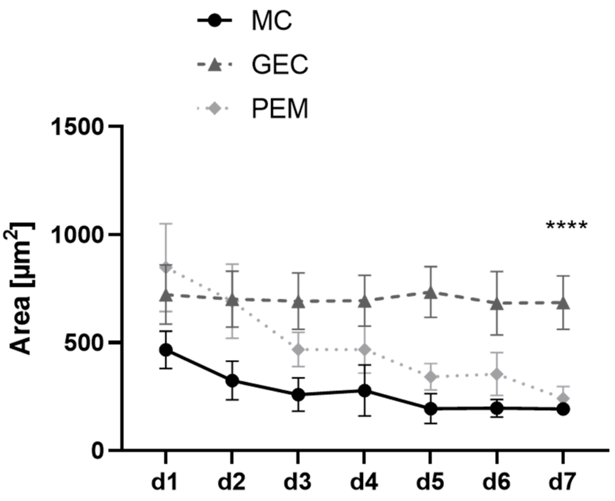

Supplementary Figure S1: *Dynamic changes of spheroid size over time.* Area of mesangial cell spheroids, glomerular endothelial cell spheroids and glomerular co-culture spheroids plotted against days. Data are presented as mean ± SD (n = 10). Data are given as mean ± SEM, **** $p < 0.0001$ GEC compared to MC or PEM spheroids. MC: mesangial cells, GEC: glomerular endothelial cells, PEM: co-culture of glomerular endothelial cells, mesangial cells and differentiated conditionally immortalized human podocytes.

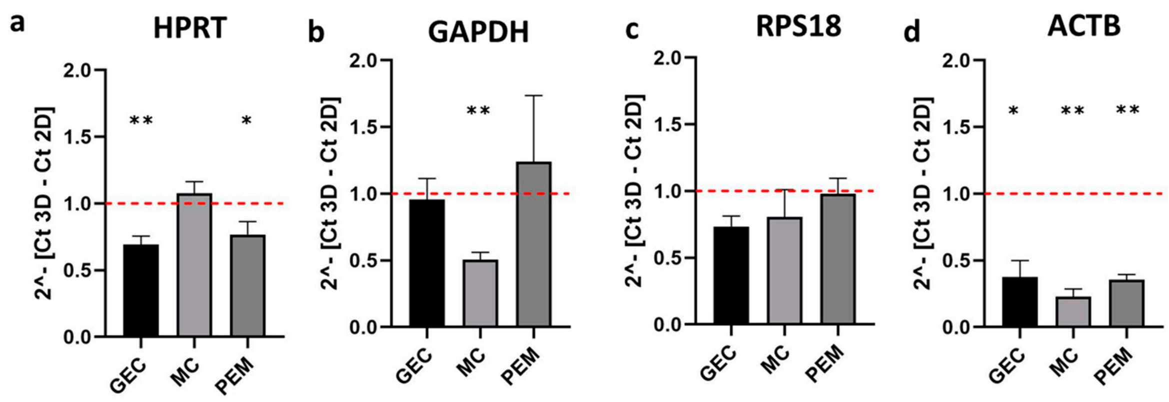

Supplementary Figure S2: *Comparison of gene expression of common housekeeper genes*. Comparison of gene expression of *HPRT* (**a**), *GAPDH* (**b**), *RPS18* (**c**) and *ACTB* (**d**) in 3D versus 2D monoculture glomerular endothelial cells and mesangial cells and co-cultures of glomerular endothelial cells, mesangial cells and podocytes**.** Data are given as fold change of 3D condition compared to 2D. n = 20 for *HPRT*, n = 4 for *GAPDH*, n=3 for *RPS18* and *ACTB*. Data are given as mean ± SEM, * $p < 0.05$, ** $p < 0.01$.

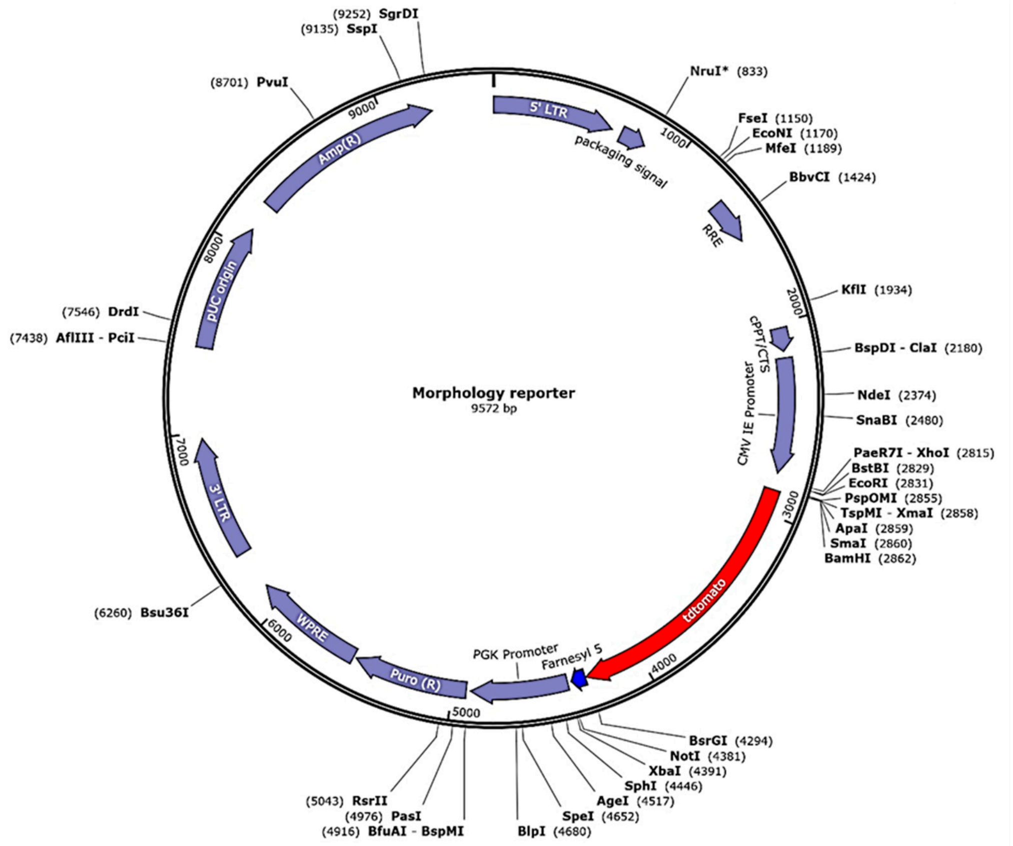

Supplementary Figure S3: *Plasmid map of morphology reporter.* Plasmid map of lentiviral transfer vector that carries the sequence for a tdTomato protein with an additional farnesylation signal. The farnesylation of the tdTomato protein leads to its incorporation into the plasma membrane and makes it possible to assess cell morphology. *: This site is blocked by Dam methylation.

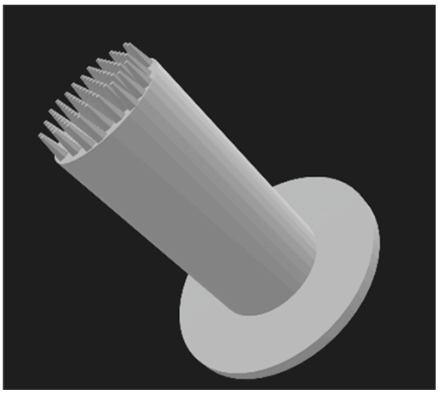

Supplementary Figure S4: *Negative mould of the agarose micro-wells.* Computer aided design projected model for moulds with 131micro-wells. The negatives are 3D-printed from resin FotoDent®. The radius of the 131 micro pillars is 1 mm each at the widest position.
